# Stakeholder and public participation in river basin management—an introduction

**DOI:** 10.1002/wat2.1086

**Published:** 2015-04-17

**Authors:** Gemma Carr

**Affiliations:** ^1^ Centre for Water Resource SystemsVienna University of TechnologyViennaAustria

## Abstract

Participation of the public and stakeholders in river basin management is increasingly promoted because it is expected to improve resource management and enable participants to engage freely and equally in management (support democratic processes). Three overlapping and interacting mechanisms by which participation is expected to enhance river basin management are outlined: (1) providing space for deliberation and consensus building for better quality decisions, (2) mobilizing and developing human and social capital for better quality decisions and their implementation, and (3) raising the legitimacy of decisions to facilitate their implementation. There are several complexities associated with each of the mechanisms that add challenges to realizing the expectations of participation. They include the need to carefully manage consensus building and conflict to maximize the quality of the decision without jeopardizing the potential for implementation; being aware of and implementing strategies to manage asymmetrical power relationships between participants; ensuring that participants perceive benefits from participation that exceed costs; and defining criteria for a legitimate process, and a legitimate decision, that satisfy all participants. Strategies identified to address these challenges focus on managing the characteristics of the participation process. Ongoing evaluation during a participation program or project is essential to reflect and refine how participation is being done, to address the challenges and endeavor to achieve high‐quality decisions that can be implemented efficiently. *WIREs Water* 2015, 2:393–405. doi: 10.1002/wat2.1086

For further resources related to this article, please visit the WIREs website.

## INTRODUCTION

The Lake Ontario‐St. Lawrence river basin is home to nearly 12 million people. The highly regulated lake and river system is used by the individuals involved in commercial shipping, recreational boaters, and hydropower who worry about whether the water will be high enough at times when they need it high. Lakeside home and business owners worry about flooding and erosion from high water levels. Urban centers need flood security. And then those concerned with the environment worry that unique species are being threatened by highly regulated water levels that go against natural cycles. All of these competing interests have to be satisfied when devising a plan to manage the water levels. To address this challenge, water managers responsible for decision making in the basin chose to engage directly with the stakeholders through participatory approaches that included meetings, workshops, debates, and consultations (see Box 1 and www.ijc.org/loslr/). This is just one of many, many examples where the complexity of the human‐water system has led to a participatory approach being chosen for river basin management.

BOX 1 **LAKE ONTARIO‐ST. LAWRENCE RIVER OPERATING SYSTEM (FROM CARR ET AL.^24^)**

**Figure 1:**
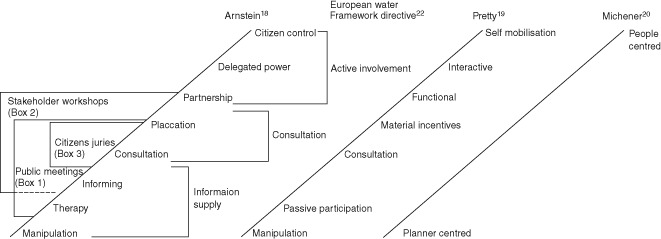
Conceptual frameworks for understanding participation based on degree of participant involvement in decision making. Types of participation described in the case studies in Boxes 1, 2, 3 are indicated.

**Figure 2:**
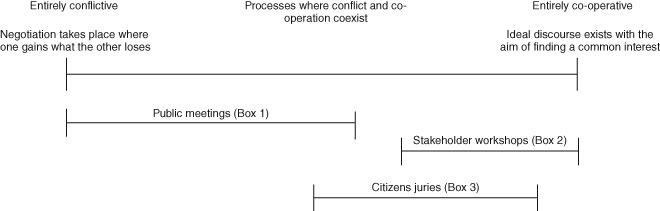
Conceptualization of participation according to whether the processes are conflictive or cooperative (based on van den Hove^23^). Types of participation processes described in the case studies in Boxes 1, 2, 3 are indicated.

The North American Great Lakes are the largest freshwater system on earth. They are also home to millions of people with many different stakes in the system, and have much endangered natural ecology. A major review of the Lake Ontario and St. Lawrence River water level operating system (the LOSL Study) was commissioned in 1999 by the International Joint Commission. The goal of the 5‐year LOSL Study was to produce an operating policy for the system that was acceptable to everyone impacted by the water levels and flows in that region. Public meetings played a significant role in providing the public and stakeholders with information on the needs and challenges of the water system, and for giving people space to voice their needs and concerns with any new lake level operating policy (see Figure 1). The LOSL Study produced three potential management plans but the polarized positions of the interest groups meant that a conflictive setting with negotiation type processes often dominated the meetings and workshops (see Figure 2) and a consensus option was not identified. The LOSL Study did however produce many other achievements beyond its original aims. Participant involvement in the development of the operating plans led them to be more innovative and raised their legitimacy, particularly when they were perceived to be supported by sound science. Awareness and understanding of other stakeholder interests also appears to have become higher amongst many participants. Recommendations to improve institutional processes, and to make water‐management decision making more representative also emerged from the LOSL Study.

Participatory approaches, defined broadly for this overview as activities that engage the public and/or stakeholders, are often encountered in river basin management. They have gained more prominence over recent decades as it has been increasingly recognized that engineering and regulation‐based management strategies are limited in their capacity to manage water resources in a sustainable way.^1^, ^2^ Proponents for participation in water resource management argue that only by moving away from a top–down management model in which decisions are made by a small group of elite professionals who are detached from the people who live and work in the basin, toward a participatory model that captures the diversity of understandings and interests in the basin, can more ethically sound and equitable management strategies be identified and employed.^1^, ^3^, ^4^, ^5^

In this context of sustainable development, participatory approaches are particularly suitable for water management. First, many different interests and conflicting objectives often coexist within a river basin, from demands by different users on water quality and quantity to flood risk and ecological health. These differences need to be addressed in order to reach a decision and bring about actions.^6^ Second, participatory approaches may help reach a decision that is perceived to be fair and legitimate, i.e., citizens are satisfied with the justification for the decision.^7^ This may be particularly important for publicly owned state managed resources such as water. Third, river basin management is information intensive because it requires knowledge of the complete socio‐ecological system, which is fragmented across many different individuals, groups, and agencies operating at different scales.^8^ By bringing ‘knowledge holders’ together with participatory approaches, this information can be mobilized and integrated into management strategies.^9^

Policy documents have high expectations from public and stakeholder participation for maintaining and improving water resources. The European Water Framework Directive^10^ states that its success in improving the aquatic environment, ‘relies on close cooperation and coherent action at Community, Member State and local level as well as on information, consultation and involvement of the public, including users’ (paragraph 14). In the United States, the Federal Clean Water Act^11^ expects participation to contribute to developing, revising, and enforcing regulations and plans (SEC. 101, e). The European Floods Directive^12^ also states that interested parties should be encouraged to be actively involved in developing flood risk management plans (Article 10).

These policies place weight on public and stakeholder participation for two reasons. First, participation is expected to enhance resource management (pragmatic reasons) and second, it should support democratic processes and enable individuals and/or groups to engage freely and equally in the management process (normative reasons).^13^, ^14^ This overview will explore the rationale for these expectations and identify the challenges in realizing the potential of participation. Using examples primarily from Europe and North America, it aims to give water resource researchers and practitioners some background on what participation is, why it is expected to support river basin management, and which strategies can help achieve these expectations.

## DEFINING AND CONCEPTUALIZING PARTICIPATION

Defining participation is not straightforward. Many words are associated with it—collaboration, deliberation, involvement, social learning, engagement, and co‐management. Several authors have suggested that it is more appropriate to think of it as a principle, rather than to define it rigorously.^15^, ^16^ This overview uses a very broad definition of involvement in a process. Those involved (the actors) may be members of the public, institutional decision makers, or individuals or representatives of groups with a specific interest in how the river basin is managed or capacity to influence the outcome (stakeholders). It has been suggested that the general public should be engaged on topics of broad public concern, such as government spending on environmental monitoring, whereas stakeholders should be engaged on specific issues, such as residents in a polluted catchment.^17^ Position and power in deciding and implementing a management strategy vary between the actors. The process may be passive, such as receiving information, or it may be active, such as contributing to planning and decision making.

Several frameworks conceptualize participation according to the degree of participant involvement in decision making (Figure 1). Each rung of Arnstein's ladder of participation indicates greater citizen involvement in the decision‐making process.^18^ This framework, developed in the field of urban planning, has strongly shaped the conceptualization of participation in other fields, including water management. From the development literature, Pretty's^19^ typology classifies participation according to the participants' extent of involvement in activities and control over outcomes. Pretty's participation ranges from manipulative, where unelected and powerless representatives sit on official boards, through to self‐mobilized where participants take initiatives and retain control over resource selection and allocation. In a similar manner, Michener^20^ classifies participation according to whether it is planner‐centered or people‐centered. Arnstein's Ladder has been further extended by Fung^21^ who conceptualizes participation in three dimensions that cover those who participates (e.g., elected officials, state administrators, professional stakeholders, lay stakeholders, and public), how they communicate (provide technical expertise, deliberate and negotiate, and express preferences), and how much authority and power they have (direct authority, co‐governance, advise and consult, communicative influence, and personal benefits).

The European Water Framework Directive^10^ considers participation to occur in three principal ways, information supply, consultation, and active involvement.^22^ Information supply includes activities that provide information to participants such as results, progress updates, and proposed plans. For consultation, people are asked to comment on issues or plans, either verbally or in writing (see Box 1). Active involvement is used to describe activities in which participants contribute actively to the water‐management process and refers not only to the involvement of the public or stakeholders, but also the involvement of government agencies or coordinating bodies. Approaches include multi‐stakeholder workshops (see Box 2), water users associations (farmer run participatory institutions to manage agricultural water use), or multi‐stakeholder platforms (management committees, citizen juries, advisory committees, and stakeholder partnerships; see Box 3).

BOX 2 **MULTI‐STAKEHOLDER WORKSHOPS FOR RIVER RESTORATION, UK (FROM PETTS)^62^**

An ecologically poor urban river in Birmingham, UK, was the focus for a European funded river restoration project. It aimed to develop and apply an approach for improved land‐use planning that incorporated active involvement of the public. A collaborative approach was selected that brought engineering experts and local public interest groups together through a series of workshops to co‐create a river restoration plan. At the workshops, the group identified how they envisioned the ideal urban river environment. For the public, the values included emotional characteristics such as tranquil, relaxing, natural, and light. For the engineering experts, values such as variety of wildlife, safe and flood free, and varied shape and form were important. A set of 13 community criteria were created that were used as a checklist during plan development to ensure that the restoration would meet the community's wishes. The approach meant that the final plan was both technically sound and socially agreeable—incorporating community values and engineering and environmental needs—because it had been coproduced. The author describes the setting as being cooperative (Figure 2), where information exchange between participants almost creates an environment of partnership (Figure 1). Several features of the participation process were noted by the author as being important for the success of the project. These included broad representation, impartial facilitation, finding common values that all understand and agree with and ensuring the information provided is understandable and accessible.

BOX 3 **CITIZENS JURIES IN THE NETHERLANDS (FROM HUITEMA ET AL.^84^)**

During a legally required regional land‐use planning process for a province in the Netherlands, the provincial parliament who were responsible for adopting the plan, commissioned citizens juries to hear the views of expert witnesses from different stakeholder interest groups and make recommendations. Three juries took place over 7 months, each composed of 12–14 local residents who were representative of the general public in terms of gender, age, and education level. Their task was to discuss the issues described by the expert witnesses, to reach a common position and develop recommendations to provide to the parliament, a process that most likely involved cooperation and negotiation between the jurors (Figure 2). The authors report how the elected officials considered the jury recommendations as support for draft policies, but did not base final policy on them (the process therefore could be described as predominantly consultative, Figure 1). Nonetheless, the final land‐use plan approved by the parliament was noted to be very similar to that recommended by the citizens juries.

A different type of conceptualization is proposed by van den Hove^23^ based on the type of interaction taking place between participants, from entirely cooperative to entirely conflictive (Figure 2). Entirely cooperative means a situation exists where participants work to find a common interest. Entirely conflictive means that negotiation takes place, whereby one participant gains what the other loses. She proposes that most participation activities take place in the space between, where cooperation and conflict coexist to varying extents.

The types of participation described in the case studies in Boxes 1, 2, and 3 are indicated in Figures 1 and 2 based on the descriptions given by the case study authors. The range of different types of participation processes within an individual project highlights the dynamic nature of participation. Processes and activities cannot be neatly assigned to one point in either of these frameworks, and it is also possible that they occupy more places on Arstein's Ladder than depicted, e.g., workshops, citizen juries, and meetings can all be used manipulatively. Figures 1 and 2 illustrate that participatory approaches occupy different places on the conceptual frameworks, often at the same time, and as such, participation is highly dynamic.^16^

## EXPECTATIONS FROM PARTICIPATION

Participation is expected to enhance resource management through a number of different mechanisms. In this overview, they are dealt with in three groups, although they are recognized to be highly overlapping and interacting, (1) providing space for deliberation and consensus building for better quality decisions, (2) mobilizing and developing human and social capital for better quality decisions and their implementation, and (3) raising the legitimacy of decisions to ‘smooth the way’ for their implementation. Better quality decisions in this overview are considered to be those that support sustainable development, and as such address social equity, economic growth, and environmental protection within river basin management in a balanced manner.^25^

### Making Space for Deliberation

Many river basin management problems can be described as ‘wicked’^26^. This term was coined by Rittel and Webber^27^ to describe planning problems that can never be completely solved. As such they have no clear formula for solution and no clear solution. Rittel and Webber^27^ propose a model of planning that ‘should be based on an argumentative process in the course of which an image of the problem and the solution emerges gradually among the participants, as a product of incessant judgement, subjected to critical argument’ (p. 162). Participatory approaches aim to facilitate this argumentative process by bringing together a wide variety of people with different interests and opinions and supporting them in identifying their own positions and those of others, leading them to learn from one another and develop a deeper understanding of the issues.^17^, ^28^ Participation, in this context, is based on Habermas' theory of rational discourse where reaching agreement is part of a utopian ideal speech situation in which all people are free to speak and question each others' assumptions and power differences do not exist, leading to a new, more robust knowledge.^29^, ^30^, ^31^ As such, decision making that includes the knowledge and perspectives of all participants is expected to produce high‐quality decisions in an efficient way,^32^ i.e., the benefits in terms of the quality of the decision exceed the costs of engaging the participants.

Social learning (also known as shared, communicative, transformative, collective, or collaborative learning) is a concept that builds on deliberation. Reed et al.^33^ emphasize that social learning is a process during which social interactions between people lead to a change in an individual's understanding of an issue or topic, and that this change also permeates beyond the individual to bring about change in a wider group of people. Other researchers describe the process of social learning as interactions and communications between people to reach a common understanding, collectively orientate toward shared interests and achieve concerted action (bringing different roles together to achieve some common end that emerges during the process).^19^, ^34^, ^35^, ^36^, ^37^, ^38^ Social learning can be thought as being closely aligned with knowledge coproduction whereby certified scientists and members of the lay public learn together and from each other to create joint knowledge about the river basin system that leads to strategies and plans acceptable to all.^39^, ^40^

### Developing and Mobilizing Human and Social Capital

Extensive human capital can be leveraged in the process of bringing people together to discuss river basin management. The variety of knowledge and skills (human capital) held by the participants can also lead to different, perhaps more creative and effective plans and strategies being identified.^28^, ^41^, ^42^, ^43^ Social capital describes the procedures, rules, and networks as well as the values and beliefs of the people involved.^44^ Higher social capital can facilitate and enable collaboration and collective action to achieve shared objectives.^19^, ^44^, ^45^ Participation programs can both create new social capital by developing networks and trust between participants^46^ and funnel existing social capital toward an issue. They can distribute responsibilities, raise societal commitment toward river basin management and facilitate implementation.^19^, ^47^, ^48^ However, in some situations, social capital can negatively affect river basin management by reinforcing unsustainable management practices.^49^ For example, strong clientelistic networks may block implementation of strategies that can enhance environmental conditions^50^, ^51^ and corruption may undermine regulation.^52^

### Legitimate Decision Making

Decisions and management strategies made through transparent, democratic, accessible processes that have included a fully representative group of participants may be viewed to be more legitimate and fair.^31^, ^53^, ^54^ In a legitimate decision, the participants feel that their input has influenced the decision, or believe that the decision has been made through a fair process, which was clearly defined and agreed upon early in the program. Decisions viewed to be more legitimate are expected to meet less resistance and therefore be easier to implement.^47^ Some empirical work supports this expectation. For example, Newig and Fritsch^55^ showed that decisions taken through participatory approaches can lead to reduced litigation rates and enhanced compliance.

## OVERCOMING THE COMPLEXITIES OF PARTICIPATION

In river basin management there are many people, with many different priorities and opinions, superimposed on nonstationary environmental conditions in which feedbacks and interactions are often unknown. As such, realizing the expectations of participation can be challenging. This section explores some of the complexities and identifies some strategies to address the challenges (summarized in Table 1).

**Table 1:** Challenges, Complexities, and Strategies for Realizing the Potential of Participation

Challenges	Strategies
*Deliberation and consensus building*	
The consensus decision should be a high‐quality decision.	Include all available knowledge^32^ through broad representation^42^ and ensure all participants have access to all knowledge (both physically and cognitively).^31^, ^56^ The process is unconstrained by available time and content that can be covered (all assumptions can be questioned).^31^, ^56^ Facilitation is impartial and highly skilled to create a ‘space of exchange’.^31^, ^53^, ^57^
Conflict between interest groups should raise the decision quality without inhibiting cooperation (implementation).	Include a cooperation rewards scheme.^42^
Endeavor to balance power between interest groups.	Include formal procedures to distribute power fairly.^48^ Train and empower low‐power individuals and groups.^31^, ^58^
*Human and social capital*	
The costs (time, money, and risk) should be balanced by the benefits of participation.	Participants should perceive that the benefits gained from participation exceed the costs.^59^ Participants should perceive that the costs of running the program are balanced by the importance of the issue being addressed.^60^, ^61^
*Legitimate decisions*	
Decisions should be viewed as legitimate.	Participants should define the criteria for a legitimate decision‐making process.^53^ Decisions should be based on knowledge that has been coproduced between scientists and stakeholders.^40^, ^62^ Full representation of the variety of values, opinions, and positions should be achieved.^61^, ^62^, ^63^

### Quality of Consensus Decisions

In river basin management, participation approaches are often expected to go beyond simply reaching consensus, to reaching the ‘right’ consensus—one that enables better decisions. Deliberation is anticipated to lead to decisions to which all parties consent. However, this may lead to an agreeable decision rather than a quality decision.^64^ This ‘lowest common denominator’, decision may meet the needs of no interest group, or be bad for non or under‐represented interest groups such as the environment or future generations.^64^, ^65^ Hedelin^32^ notes that just because there is agreement on the decision, does not mean that it is the right decision in relation to all the available knowledge. This highlights the importance of ensuring all available knowledge is included and accessible to participants. Others have emphasized that the features of the processes are critical for the quality of the decision. Amason's^42^ review shows that participant diversity raises the potential for high‐quality decisions, but for this potential to be realized the process needs to, ‘identify, extract and synthesize [participants] perspectives to produce a decision’, (p. 124). Broad representation is essential to ensure as much available knowledge as possible is included. The process must then focus on synthesizing this knowledge through impartial and highly skilled facilitation that ensures a ‘space of exchange’ is created where participants can speak openly and honestly.^31^, ^53^, ^57^

Achieving representation within an activity may not be straightforward. There is a risk that communities may be considered as homogenous groups, which ignores the differences in views, knowledge, experiences, and influence of the individuals.^66^, ^67^ Bringing a representative collection of participants into a process may also be challenging. Participants may become fatigued by volunteer activities^68^, or lose interest in participating if they have been regularly solicited to take part in surveys, interviews, or other activities. Byron and Curtis'^68^ research into burnout in volunteers tackling land and water degradation in Australia shows that achievable objectives and measurable intermediate indicators that demonstrate successes (such as agreement on a plan or a shared goal and vision) help achieve positive long‐term engagement.

In many situations, consensus may appear unachievable because a common interest may not exist.^30^, ^32^ Some authors have suggested that in such cases, participatory approaches would need to focus on facilitating negotiation and identifying appropriate penalties and incentives rather than enabling deliberation.^36^ Others have drawn attention to the importance of conflict for achieving high‐quality decisions.^23^, ^31^, ^42^ Hillier^30^ suggests that in a conflict situation planners should identify who is not happy and who is excluded and work to bring these people more intensely into the discussions. Amason^42^ finds that attempts to minimize conflict can lead to lower quality decisions that are easier to implement. He suggests a trade‐off occurs—on one hand, conflict is good as the decision reached will be better, on the other hand, conflict means the possibilities of reaching a decision that can be implemented are lower. However, conflict appears to occur in two very closely related forms. Cognitive conflict, which is focussed on differences in judgments, raises the decision quality without eroding the potential for cooperation in implementation. Affective conflict, which is emotional and focussed on personal disputes, prevents consensus being reached and implementation taking place. To address this, he suggests placing mechanisms such as a cooperative rewards scheme prior to initiating a conflict situation to endeavor to keep conflict cognitive rather than affective.

Unequal power between participants is an inherent feature of any participation process.^30^, ^31^, ^32^ Deliberation and consensus building may be used by powerful interest groups to give the impression of sharing power, while actually using the process to ensure they get the decision they want.^30^, ^69^, ^70^ Manipulation of participation processes may manifest through exclusion of certain participants, or their inclusion with only limited ‘voice’ in the discussions.^70^ Such manipulation will inhibit achieving a high‐quality decision according to all available knowledge. Hemmati^48^ suggests that formal procedures can help to overcome social power imbalances, and Leach et al.^58^ draw attention to the need for projects and policies that prepare and empower disadvantaged groups to better argue their case against more powerful actors. Knowledge that more closely conforms to Western‐science and rationality is typically perceived to have more value than indigenous or local knowledge.^67^ This means that either indigenous knowledge holders must find ways to articulate their knowledge in a way that conforms to Western knowledge systems, or the participatory process must endeavor to give ‘voice’ to indigenous or local people to ensure that their opinions truly challenge Western‐centric scientific world views and propose alternative agendas and strategies.^67^

### Costs and Benefits

Participatory approaches can have higher financial costs than top–down decision making as they require extra personnel and administration, they are time demanding and can delay decision making.^71^ As such, their efficiency can be brought into question if an expensive participatory process reaches the same decision that would have been made by a single decision maker, or if the decision maker ignores the contribution from participants.^72^ Another concern is that citizens may make choices that are either too expensive or impractical to implement.^14^, ^43^ Cost‐effectiveness can therefore mean several things: (1) that the costs of running a participation program are matched by the importance of the decision being made,^60^, ^61^ (2) that the benefits to participants are perceived to exceed their costs,^59^ (3) that the decision that results is of lower cost than that which would emerge without participation,^60^ or (5) the decision that results has lower implementation or enforcement related costs.^73^

Empirical work on cost‐effectiveness in participatory decision making has led to the suggestion that more intensive processes that run for longer, are well funded and attract committed participants create a positive setting for creative decision making that can lead to more cost‐effective solutions being found.^43^ Box 2 illustrates a collaborative planning exercise where workshops led to the coproduction of a plan that met the needs of the public and engineers.

Participating may present risks to some individuals or interest groups if the program does not lead to intended outcomes or if it causes harm to some interests.^59^, ^70^ Participants must therefore perceive that they will gain benefits from their involvement that outweigh any potential risks. Benefits can be very broad and may range from tangible outcomes, such as an agreement and associated support that leads to action, to intangible outcomes such as the development of networks, friends, trust, cooperation, or learning.^74^ (See Box 1).

### Defining a Legitimate Decision

The typologies of participation (Figure 1) suggest that approaches higher up the ‘ladder’, those that provide participants with more capacity to influence decisions, are the ultimate achievement. Several researchers have contested this conceptualization with arguments of whether citizens who hold no formal training in the field, legal authority, or accountability, should be empowered to take decisions.^28^, ^75^ Renn et al.^14^ emphasize that legitimate decision makers are those elected through due process and public approval and whereas citizen participation has a place for making recommendations, legitimate power holders should make the final decision. While Gooch and Huitema^76^ claim that there are few decisions in practice made solely by elected representatives because government agencies and experts, pressured by organized lobby groups, strongly influence decisions. Participation, they propose, can make this process more transparent. Some of the features of legitimate decision making are that it is based on evidence rather than political motivations^16^ though researchers have drawn attention to the highly political nature of science^77^ and the way that scientifically derived knowledge and evidence can be either selected or excluded to support a favored plan or decision.^78^ The coproduction of knowledge between ‘certified’ scientists and lay stakeholders not only aims to capture and harmonize a wide variety of understandings and knowledge leading to ‘one collective knowledge’ that is trusted by all, but the process also bring interests and agendas to the surface where they can be more openly addressed.^40^, ^79^, ^80^ Whichever way decision‐making functions, what is noted as critical for a legitimate process, is that it is clearly structured and clearly displayed. 61, 81 Rowe and Frewer^61^ highlight that participants need to have a clear understanding and agreement on how their input will be used (e.g., to directly contribute to the final decision, or to bring issues to light to those who will make the final decision; see Box 3).

In some situations, governments may engage in a participatory approach for politically difficult decisions for which they wish to remove their culpability.^70^ Participation is also noted to be used as a rationale by which governments can transfer financial and administrative responsibilities to citizens.^20^ For example, in response to spending cuts, the UK government proposed the ‘Big Society’ whereby citizens undertake voluntary work to ensure that previously funded areas, such as environmental monitoring, continue to take place.^82^ The normative democratic aspect of participation suggests that participants should be involved in the decision making on whether a participatory process is used for decision making, and if so, how that process should look, i.e., who is responsible for the decision and management.

Representativeness of the participants is another feature of legitimate decisions that, as described earlier, is highly complex. Stakeholders or citizens (including those who do not directly take part in the participation activity) need to perceive that their values, opinions or positions have been represented within the process. The representativeness of the participants involved in a process can often be contested. Identifying and bringing a collection of individuals who represent the full spectrum of opinions and positions into the process is particularly challenging in river basin management where the issues being addressed are often unknown or uncertain at the start of the process. Stakeholder analysis by the agency initiating the participatory approach may accidently or intentionally fail to identify some stakeholders leading to their subsequent exclusion. Those who are brought into a process may have conflicting claims to the constituencies or positions they allege to represent, and may have different levels of authority to represent their constituencies. Blackstock et al.^63^ show how the organization representatives in advisory groups for Scottish river basin planning often showed affiliation to more than one constituency during the process, e.g., industry representatives may also be keen fishermen or naturalists. While representatives from some groups chose not engage in the advisory groups, but relied on alternative approaches such as one‐on‐one meetings with agency staff to lobby for their interest group. Lamers et al.^83^ describe how the participatory approaches employed to develop a water‐management plan in the Netherlands led to strategies being incorporated to which one group of stakeholders (tenant farmers), who had not been included in discussions, were fiercely objected at the end of the process. Lamers et al. suggest that during the participation process, the problem framing (i.e., what issues are problems, who might be responsible for them and what should be done about them) evolved but the participant representation remained the same. Both Lamers et al. and Blackstock et al. emphasize that representation is not static, and must be continually sought throughout any participatory project or program.

## ONGOING EVALUATION

There is considerable diversity in the challenges and strategies described above and outlined in Table 1. However, there is one aspect in common throughout. This is that ongoing evaluation is essential to assess which strategies are needed, whether they are being implemented, and if they are having the desired effect for overcoming the challenges. As such, evaluation, reflection, and refinement of an ongoing participation program are critical to overcome the complexities of doing participation and realizing the expectations of deliberation and consensus building, developing human and social capital, and producing legitimate decisions that are the foundations for high‐quality decisions that can be implemented effectively.

Much work has been conducted to evaluate participation processes relative to whether different objectives are met, such as whether a deliberative environment is created^75^, whether the process meets the participants' expectations^85^, or whether participants accept the decisions reached.^61^ The large body of work has identified several important characteristics of good processes for participation programs and projects that are included in the strategies in Table 1. Of particular importance are impartial facilitation with a highly skilled facilitator, creation of a space of exchange where all participants have the opportunity to be heard, participant access to information and meetings, clearly defined tasks and ground rules for interaction, and representation of a broad section of society/interest groups.^53^ (See Box 2).

Evaluating outcomes is important for identifying whether participation is leading to high‐quality decisions and what impacts they have on river basin management. However, defining outcomes is dependent on interest. Different people have very different objectives from a participation program.^20^, ^53^ Researchers interested in identifying whether participation improves ecological conditions will focus on environmental outcomes such as ecosystem health or water quality. Water managers may be interested in evaluating whether a participation program is leading to a reduction in conflict between interest groups, and agriculturalists might look for economic benefits such as an increase in crop yields. Participants may think the program is a success if they are satisfied with how it has been run^86^, or if they see tangible outcomes (such as an agreement that supports their interests), or intangible outcomes (such as a change to institutions, development of shared knowledge, extension of personal networks, or learning about new issues) (see Box 1).^74^ Evaluation of these intangible, intermediary outcomes has been suggested as a means to assess what participation is achieving that overcomes the difficulties of both identifying and measuring resource management outcomes.^53^

## CONCLUSIONS

This overview has aimed to give an introduction to stakeholder and public participation. A participatory approach in river basin management is promoted for normative reasons, that people should have the opportunity to contribute to environmental management freely and equality, and pragmatic reasons, that it is expected enhance resource management by leading to better quality decisions that support sustainable development and can be efficiently implemented. In this overview, the many mechanisms by which these outcomes are expected to be achieved have been coarsely grouped according to deliberation and consensus building, developing and mobilizing human and social capital, and raising the legitimacy of decisions.

Several complexities and challenges in realizing the expectations of participatory programs or projects have been identified through the literature. They include: (1) carefully managing consensus building and conflict to maximize the quality of the decision without jeopardizing the potential for implementation, (2) being aware of and implementing strategies to manage asymmetrical power relationships between participants to maximize the quality of the decision, (3) ensuring that participants perceive benefits from a participation program that exceed the costs of running the program, and (4) defining criteria for a legitimate process, and a legitimate decision, that satisfies all participants. Strategies to address these challenges are focussed around how the participation processes are managed. This suggests that ongoing evaluation during a participation program or project is essential to reflect and refine how participation is being done, to overcome the challenges and to ultimately achieve high‐quality decisions that can be implemented efficiently.
